# Mechanical Properties, Drug Release, Biocompatibility, and Antibacterial Activities of Modified Emulsified Gelatin Microsphere Loaded with Gentamicin Composite Calcium Phosphate Bone Cement In Vitro

**DOI:** 10.3390/ma17143578

**Published:** 2024-07-19

**Authors:** Ming-Hsien Hu, Bo-Sin Shih, Shih-Ming Liu, Ssu-Meng Huang, Chia-Ling Ko, Wen-Cheng Chen

**Affiliations:** 1 Orthopedic Department, Show Chwan Memorial Hospital, Changhua 500, Taiwan; minghsienhu@gmail.com; 2 Department of Post-Baccalaureate Medicine, College of Medicine, National Chung Hsing University, Taichung City 402, Taiwan; 3 Advanced Medical Devices and Composites Laboratory, Department of Fiber and Composite Materials, Feng Chia University, Taichung City 407, Taiwan; a12345935661@gmail.com (B.-S.S.); 0203home@gmail.com (S.-M.L.); dream161619192020@gmail.com (S.-M.H.); rayko1024.rb@gmail.com (C.-L.K.); 4 School of Dentistry, College of Dental Medicine, Kaohsiung Medical University, Kaohsiung 807, Taiwan; 5 Department of Fragrance and Cosmetic Science, College of Pharmacy, Kaohsiung Medical University, Kaohsiung 807, Taiwan

**Keywords:** calcium phosphate bone cement (CPC), emulsification, hydrogels, microspheres, antibiotic, in vitro, composite

## Abstract

Bone defects are commonly addressed with bone graft substitutes; however, surgical procedures, particularly for open and complex fractures, may pose a risk of infection. As such, a course of antibiotics combined with a drug carrier is often administered to mitigate potential exacerbations. This study involved the preparation and modification of emulsified (Em) crosslinking-gelatin (gel) microspheres (m-Em) to reduce their toxicity. The antibiotic gentamicin was impregnated into gel microspheres (m-EmG), which were incorporated into calcium phosphate bone cement (CPC). The study investigated the effects of m-EmG@CPC on antibacterial activity, mechanical properties, biocompatibility, and proliferation and mineralization of mouse progenitor osteoblasts (D1 cells). The average size of the gel microspheres ranged from 22.5 to 16.1 μm, with no significant difference between the groups (*p* > 0.05). Most of the oil content within the microspheres was transferred through modification, resulting in reduced cytotoxicity. Furthermore, antibiotic-impregnated m-EmG did not compromise the intrinsic properties of the microspheres and exhibited remarkably antibacterial effects. After combining with CPC (m-EmG@CPC), the microspheres did not significantly hinder the CPC reaction and produced the main product, hydroxyapatite (HA). However, the compressive strength of the largest microsphere content of 0.5 wt.% m-EmG in CPC decreased significantly from 59.8 MPa of CPC alone to 38.7 MPa of 0.5m-EmG@CPC (*p* < 0.05). The 0.5m-EmG@CPC composite was effective against *Staphylococcus aureus* (*S. aureus*) and *Escherichia coli* (*E. coli*) in drug release and antibacterial tests. Compared with m-EmG alone, the 0.5m-EmG@CPC composite showed no toxicity to mouse fibroblast cells (L929). Additionally, the proliferation and mineralization of mouse osteoblastic osteoprogenitor cells (D1 cells) did not have a negative impact on the 0.5m-EmG@CPC composite over time in culture compared with CPC alone. Results suggest that the newly developed antibacterial 0.5m-EmG@CPC composite bone cement did not negatively affect the performance of osteoprogenitor cells and could be a new option for bone graft replacement in surgeries.

## 1. Introduction

In today’s clinical operations, bone repair surgeries are often treated with bone graft substitutes. Bone substitutes can generally be divided into two categories: ceramic-based and polymer-based. Ceramics come in different forms, such as block, granular, cement, etc. Most notably, calcium phosphate (CaP) ceramics are chemically very similar to bone minerals, are biocompatible, and are now widely used clinically as CaP has been shown to be reliable and safe in bone regeneration applications [1,2,3]. Bone substitutes can also be divided into autologous grafts, allogeneic grafts, and synthetic bone substitutes, according to the source of the grafts [4]. Of the three categories, synthetic bone substitutes have better biocompatibility and mechanical properties than the two other bone grafts and are chemically similar to human bone. In addition, composite bone substitutes can even be adapted by bone tissue for resorption and regeneration. Osteoconductive prostheses reduce the risk that a second surgery may be required, which may reduce postoperative infection. These advantages allow the reuse of synthetic bone substitutes in clinical applications [5,6,7].

Calcium phosphate bone cement (CPC) is a slurry of powder mixed with a self-hardening solution that reacts to form hydroxyapatite (HA). It is applied as a bone-filling material and has excellent biocompatibility, osteoconductivity, injectability, and plasticity. However, traditional CPC has shortcomings, such as insufficient mechanical strength, long hardening time, and difficulty in controlling the final product phase [8,9,10]. Therefore, many strategies have been developed by combining it with other medical devices to modify its performance and provide versatility [11,12,13].

Oral or intravenous antibacterial or anti-inflammatory drugs are often given during the recovery and treatment of complex fractures. However, the effective absorption of drugs is extremely low and often cannot effectively reach the affected area. To improve these shortcomings, drug carriers are used to efficiently improve the body’s absorption of drugs, accurately deliver drugs to the recovery area, and reduce the dosage and the risk of side effects [14,15,16,17,18]. Studies have confirmed that drugs combined with acellular perichondrium hydrogel can be antibacterial and effectively promote bone repair [19].

Hydrogel-based microspheres are commonly used as drug carriers. Various preparation strategies include freeze-drying, freeze-sublimation of lyophilization, emulsification, electrospray, and 3D bioprinting. Hydrogel microspheres prepared by emulsification have a relatively simple process and a low cost. In addition, the microsphere structure is relatively stable, and the drug release and degradation cycle is long. Therefore, this method was chosen to prepare microspheres in the present experiment [20,21,22].

Emulsification combines two immiscible phases, such as oil and water, by using surfactants (emulsifiers) to create a uniform dispersion or emulsion. This study implemented a water-in-oil (*w*/*o*) emulsification method to produce hydrogels. In microsphere manufacturing, gelatin (gel) serves as the aqueous phase, while paraffin oil is utilized as the oil phase. Gelatin is a hydrolyzed derivative of collagen and is generally derived from the skin, bones, and connective tissue of animals. It has good degradability, plasticity, and non-toxicity and is often used in the fields of food and tissue engineering. Gelatin is widely used to prepare drug carriers because of its easy-to-form adhesive properties [23,24]. Paraffin oil, also known as liquid paraffin or white oil, is a colorless, odorless, transparent mineral oil. It is a product of the high-temperature fractionation of petroleum. Paraffin oil has good chemical stability, and fewer impurities are produced when used in the oil phase of an emulsion [25].

Span80 is a surfactant suitable for preparing without emulsified microspheres; it is a biodegradable surfactant based on natural fatty acids (oleic acid) and the sugar alcohol sorbitol [26]. Chemical cross-linking with glutaraldehyde (GA) reacts quickly with gelatin and is therefore used in this study to improve the stability of the gelatin structure. Compared with other chemical cross-linking agents, GA leads to a shortened process time. Paraffin oil, Span80, and GA have certain toxicity [27], so ammonium dihydrogen phosphate (ADP) has been added to modify gel microspheres. The phase produces a saponification reaction to achieve a washing effect, thereby removing the oil phase and excess GA cross-linking agent [28].

Gentamicin is an antibiotic with good antibacterial effects against Gram-negative and some Gram-positive bacteria. The antibacterial mechanism is that when the drug enters the human body, it will enter the interior of the bacteria through the hydrophilic channels on the bacterial surface, thereby inhibiting bacterial ribosomes from synthesizing proteins and causing bacterial death [29]. The present study aims to prepare an emulsified microsphere, which was further modified with ADP and impregnated with gentamicin to exert anti-inflammatory effects, especially for open-wound restorations. Finally, considering clinical applicability, modified gentamicin-emulsified gel microspheres composited with commercially available calcium phosphate bone cement (CPC) and their performance, antibacterial properties, and in vitro biocompatibility were characterized. In the future, it can be directly used to repair complex open bone defect wounds, shorten the inflammation time, and achieve rapid healing.

## 2. Materials and Methods

### 2.1. Raw Materials

The raw materials used in this study included the following: gelatin (type B from bovine skin, with an average molar mass of 40,000–50,000 g/mol and a protein purity specification of about 75%, Sigma-Aldrich, Saint Louis, MO, USA), paraffin oil (Macron, Avantor, Radnor, PA, USA), sorbitan monooleate (Span 80, Emperor Chemical Co., Ltd., Taipei City, Taiwan), a solution of 25% glutaraldehyde for gelatin crosslinking (GA, Panreac AppliChem, Barcelona, Spain), ammonium dihydrogen phosphate for modification (ADP, Katayama Chemical, Osaka, Japan), and gentamicin for microsphere impregnation (Siu Guan Chemical Industrial Co., Ltd., Chiayi, Taiwan). The main components of calcium phosphate bone cement (CPC) are tetracalcium phosphate (TTCP) and anhydrous dicalcium phosphate (DCPA). The source of the material is the commercial CPC of the product “REALBONE”, an absorbable calcium phosphate bone graft substitute (injectable type) provided by a domestic medical device manufacturer (Realbone Technology Co., Ltd., Kaohsiung, Taiwan).

### 2.2. Preparation of Gel Microspheres (Em, m-Em, and m-EmG) and CPC Composites (m-Em@CPC and m-EmG@CPC)

#### 2.2.1. Emulsified Gel Microspheres (Em)

The process began by mixing 1.5 g of gel with 10 mL of deionized water (diH_2_O), and the solution was heated to 60 °C to obtain a 15% (*w*/*v*) gel solution. Subsequently, 0.2 mL of Span 80 was added to 20 mL of paraffin oil, and the mixture was stirred and heated to 60 °C. About 2 mL of the gel solution was slowly dripped through a syringe into the prepared water-in-oil solution under continuous stirring for 30 min. The resulting mixture was then placed in an ice bath at 5 °C and cross-linked with 1.5 mL of GA for 30 min. After cross-linking, hydrogel microspheres were formed and then washed three times with acetone and diH_2_O to remove excess oil-phase substances. Further filtered and dried to obtain emulsified microspheres (Em).

#### 2.2.2. Em Modification (m-Em) and Antibiotic Impregnation (m-EmG)

The hydrogel microspheres were soaked in a 1 g/250 mL of ADP solution and reacted for 24 h to remove the residual oil phase and modify Em. Suctioning and filtration were performed to obtain the modified microspheres (m-Em). About 0.02 g of m-Em microspheres were taken and immersed directly in 10 mg/mL gentamicin for 24 h before being lyophilized for another 24 h to obtain m-EmG microspheres.

#### 2.2.3. m-EmG Composite CPC Process

The mixed powder of CPC consisting of TTCP to DCPA molar ratio of Ca/P was 1.67 [30]. About 0.8 g of CPC powder was mixed with 0.1 wt.%, 0.3 wt.%, and 0.5 wt.% m-EmG microspheres, followed by adding 380 μL of 0.067M dilute phosphate hardening solution to form a paste. The plate was transferred into a stainless-steel cylinder mold measuring 6 mm in diameter and 12 mm in height and then processed to remove the mold within 15 min of the initial setting. The resulting samples were immersed in the simulated body fluid of Tris buffer solution and subjected to relevant mechanical, physical, and chemical tests, along with in vitro cellular reaction tests for comparative analysis.

### 2.3. Physiochemical Properties

#### 2.3.1. Microstructure Observations

A scanning electron microscope (SEM; S-3400N, Hitachi, Tokyo, Japan) was used to analyze differences in the surface morphology of Em, m-Em, and m-EmG.

#### 2.3.2. Spectral Analysis by Fourier Transform Infrared Spectroscopy (FTIR)

The analyzed solid samples (gelatin powder, m, m-Em, m-EmG, and m-EmG@CPC) were ground and thoroughly mixed with optical-grade KBr powder at a ratio of 1/100 (*w*/*w*) for FTIR analysis. Liquid samples (paraffin oil, Span 80, glutaraldehyde, and gentamicin) were tested by reflections from the diamond surfaces using an attenuated total reflection-FTIR (ATR-FTIR; Nicolet iS5, Thermo Fisher Scientific, Waltham, MA, USA). It was scanned from 400 cm^−1^ to 4000 cm^−1^ to analyze changes in the spectrum.

#### 2.3.3. Changes in Immersion Weight of Hydrogel Microspheres

In this experiment, 0.01 g of microspheres from different groups were soaked in diH_2_O and kept at 37 °C. Excess water was removed before the samples were weighed at different time points until complete degradation. The calculation formula is as follows:

$$weight\;changes\;\left(\%\right)=\frac{W_{0}-W_{t}}{W_{0}}\times 100\%$$

where *W*_0_ is the initial weight, and *W_t_* is the weight of the sample after different immersion days.

#### 2.3.4. Crosslinking Index Changes

Ninhydrin (2,2-dihydroxy-1,3-indanedione, Sigma-Aldrich^®^, St. Louis, MO, USA) reacts with free amine groups to form a blue-purple product, with a darker color indicating a reaction. The presence of more amino groups leads to a lower degree of cross-linking. Residual amino groups of the microspheres before and after cross-linking were determined at an optical density wavelength (OD_570_) of 570 nm by using an enzyme-linked immunosorbent assay microplate (ELISA) reader (SPECTROstar Nano, BMG LABTECH, Offenburg, Germany). Fixation index was calculated as follows [31]:

$$\mathrm{Fixation}\mathrm{index}(\%)\;=\frac{{\left(\mathrm{activated}\;\mathrm{amino}\right)}_{\mathrm{fresh}}\;-\;{\left(\mathrm{activated}\;\mathrm{amino}\right)}_{\mathrm{residual}}}{{\left(\mathrm{activated}\;\mathrm{amino}\right)}_{\mathrm{fresh}}}\times 100\%$$

The free amine group before crosslinking is (amine-reactive)*_fresh_*, and the free amine group after crosslinking is (amine-reactive)*_residual_*.

#### 2.3.5. Thermal Stability

Thermogravimetric analysis and a differential scanning calorimeter (TGA-DSC; TA-MDSC, TA Instrument, New Castle, DE, USA) were employed to assess the structural stability of m-Em following drug impregnation of m-EmG, up to a temperature of 400 °C at a heating rate of 2 °C/min.

#### 2.3.6. Gentamicin Release

The measured 0.01 g of m-EmG microspheres and 1 cm cubes of m-EmG@CPC were soaked in 3 mL of deionized water (diH2O) and then tested using ultraviolet-visible spectroscopy (UV-Vis; UV1800, Shimadzu, Kyoto, Japan) to measure the release of each group. The optical density of gentamicin was recorded at a wavelength of 193.2 nm (OD_193.2_). The calibration curve formula is:

*y* = 0.002622 *x* − 0.000047.

To obtain the y value, substitute OD_193.2_ into *x* and convert it to antibiotic concentration.

#### 2.3.7. Phase Identification

The sample was analyzed using an x-ray diffractometer (XRD; XRD-6000, Shimadzu, Kyoto, Japan) to study changes in the crystallization of m-EmG@CPC composites. The diffraction conditions included Ni-filtered Cu target Kα with 30 kV and 20 mA. The scan angle ranged from 20° to 60°, and the 2*θ* scan rate was 2°.

#### 2.3.8. Working/Setting Time Measurements

During the mixing of one cement, the slurry gradually hardened without any mutual adhesion between the powders. This period is known as the working time. The setting time was determined by using a dental phosphoric acid standard, ISO 9917-1 [32]. This involved pressing down on the sample vertically with a 400 g Gillmore needle at 37 °C and 60–70% humidity until no visible indentation was found on the surface. This marked the end of the hardening period.

#### 2.3.9. Injectability and Disintegration

After mixing the composite paste for 1 min, it was immediately transferred into a 5 mL syringe. A vertical force of 250 N was applied to the syringe barrel, and the bone cement was injected into diH_2_O at 37 °C within 3 min to achieve injectability and disintegration. Pictures were captured to observe changes after the composite cement was injected into the water. If no disintegration was observed after soaking for one day, it was considered disintegration resistant.

#### 2.3.10. Compressive Strength and Fracture Surface Observation

The sample’s strength was tested in accordance with ASTM F451-16 [33]. The m-Em@CPC and m-EmG@CPC samples with a diameter of 6 mm and height of 12 mm were immersed in a Tris-buffer solution, an artificial body fluid, at a 1 g to 10 mL ratio. After being placed at 37 °C for 1 day, it was removed, and the compressive strength of the wet composite specimens was measured using a universal testing machine (HT-2402, Hung Ta, Taichung, Taiwan) at a crosshead speed of 1 mm/min. After the test, the fractured sample was immersed in ethanol to stop the reaction. The fracture surface of the m-EmG@CPC composite after high vacuum metal evaporation coating was observed using SEM.

### 2.4. Antibacterial Tests

#### 2.4.1. Qualitative Antibacterial Testing

*Staphylococcus aureus* (*S. aureus*; ATCC number: 25923) and *Escherichia coli* (*E. coli*; ATCC number: 10798) were selected for qualitative and quantitative testing. The culture environment of the strain was maintained at 37 °C without CO_2_ to avoid bacterial overgrowth. In addition, control CPC alone and experimental m-EmG/CPC composites were autoclaved before the experiments.

#### 2.4.2. Quantitative Antibacterial Testing

Antimicrobial quantification was tested using the broth dilution method. First, 0.8 g of a cylindrical sample with a diameter of 6 mm was placed in 2 mL of bacterial suspension with a density of approximately 1.0 × 10^7^ cells/mL and incubated with bacteria at 37 °C for 1 day. About 100 µL of the bacterial suspension was transferred to a 96-well plate. Absorbance (OD_595_) was recorded using an enzyme-linked immunosorbent assay (ELISA) reader (EZ Read-400, Biochrom, Holliston, MA, USA) to reflect the antibacterial effect.

### 2.5. In Vitro Cytotoxicity and Osteoprogenitor Cell Activities

#### 2.5.1. Cell Culture

Fibroblast L929 cells (National Institute of Health, Miaoli, Taiwan) were used in the study. The test followed the rules outlined in ISO 10993-5:2009 [34]. L929 cells were cultured in an incubator at 37 °C with 5% CO_2_ and subcultured at a cell concentration of 0.8–1.0 × 10^6^ cells/mL. The minimal essential medium alpha (Gibco, Thermo Fisher Scientific Inc., Waltham, MA, USA) containing 10% horse serum was used, which was changed every 2 days. Bone marrow mesenchymal stem cells were cloned from Balb/C mice with osteoprogenitor D1 cells from the American Type Culture Collection (ATCC). Short-term D1 cell attachment, proliferation, semi-quantitative, and qualitative measurements for alkaline phosphatase (ALP) were evaluated to assess the biological response between composites and D1 cells. The culture medium used was Dulbecco’s modified Eagle’s medium (Thermo Fisher, Waltham, MA, USA) containing 10% fetal bovine serum (Thermo Fisher, Waltham, MA, USA).

#### 2.5.2. Cytotoxicity Tests

According to the ISO 10993-5 specification, Em, m-Em, and m-EmG were extracted in the cell culture medium according to the ratio of 1 g/10 mL, while m-Em@CPC and m-EmG@CPC were immersed in the cell culture medium according to the extracting ratio of 1 g/5 mL. After 24 h of incubation at 37 °C, the culture medium was extracted for the cytotoxicity test. The control group consisted of a blank cell culture medium, while the positive control group was exposed to 15% dimethyl sulfoxide (DMSO; Sigma-Aldrich, St. Louis, MO, USA), and the negative control group was exposed to high-density polyethylene (HDPE). All samples used for subsequent in vitro cell culture were sterilized under γ-radiation at 25 kGy (China Biotechnology Co., Ltd., Taichung, Taiwan).

In the quantitative cytotoxicity assay, each group of extracts (100 μL/well) was added to L929 cells at a concentration of 1 × 10^4^ cells, and the cells were cultured for 24 h. After that, the medium was removed, and 100 μL of fresh medium was mixed with 50 μL of XTT cell proliferation assay kit (Biological Industries, Kibbutz Beit Haemek, Israel) for a 4-h reaction. Subsequently, the samples were processed using an ELISA reader (SPECTROstar Nano, BMG LABTECH, Offenburg, Germany). The measured absorbance from OD_492_ was directly proportional to cell viability. In the qualitative cytotoxicity, the same extract was used to culture L929 cells (1000 μL/well) with a cell concentration of 1 × 10^5^ cells for 24 h, and then the cell morphology was observed.

#### 2.5.3. D1 Cell Proliferation, Mineralization, and ALP Staining

Composite surfaces were cultured with D1 cells at a D1 cell concentration of 1 × 10^5^ cells for 1, 4, 7, 10, and 14 days to allow for proliferation. After incubation, the samples were washed with PBS, and the new culture medium was added to the detecting assays. Cell metabolic activity and ALP (an early marker of osteogenesis) were tested using an alamar Blue assay (Bio-Rad, Hercules, CA, USA) and *p*-nitrophenyl phosphate kits (pNPP; Sigma-Aldrich, St. Louis, MO, USA). The absorbance of alamar Blue at OD_570_ was directly proportional to the metabolic activity of the cells, and OD_590_ was used as the reference wavelength. The absorbance of the reaction solution was determined at OD_405_ by the measurement of the ALP amount by the ELISA reader. ALP staining was carried out using SIGMAFAST BCIP/NBT tablets (N2770, Sigma-Aldrich, St. Louis, MO, USA). The composites, after the cell culture, served as the ALP staining substrate. The composite samples were washed three times with water before being examined under a light microscope (IVM-3AFL, Sage vision Co., New Taipei City, Taiwan). A darker color indicates a higher ALP content.

### 2.6. Statistical Analysis

An Analysis of Variance (ANOVA) was conducted using IBM SPSS Statistics version 20 (SPSS Inc., Chicago, IL, USA) to assess the properties of Em, m-Em, and m-EmG microspheres and m-Em@CPC and m-EmG@CPC composites. ANOVA was utilized to determine the significance of differences among the means of multiple groups. The comparison of differences relied on the estimates of two distinct variables.

## 3. Results and Discussion

### 3.1. Analysis of Different Gel Microspheres

#### 3.1.1. Observation of Em, mEm, and m-EmG Microspheres

**Figure 1:**
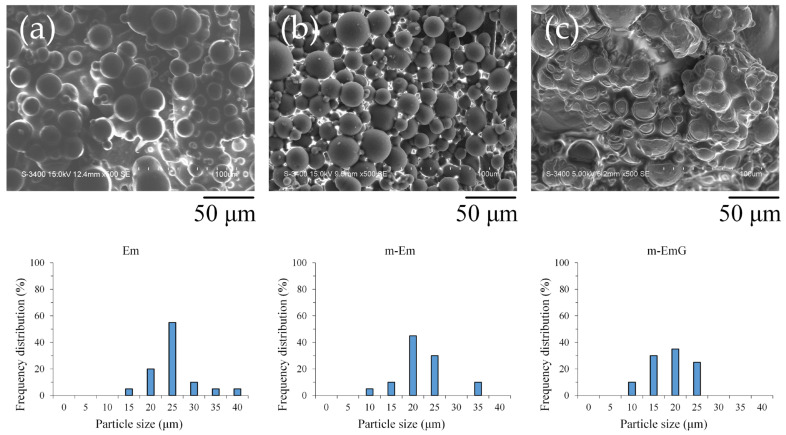
SEM microstructure of each emulsified gel microsphere (left to right: (**a**) Em, (**b**) mEm, and (**c**) m-EmG); the particle sizes of the three groups of Em, m-Em, and m-EmG are 22.5 ± 5.7, 19.6 ± 5.2, and 16.1 ± 4.8 μm, respectively (*n* = 20, *p* > 0.05).

Upon examination of the microstructure (Figure 1), it was apparent that Em before modification exhibited significant agglomeration, with most of the microspheres being coated or interlocked with the oil. However, despite the slight agglomeration in m-Em, it was discernible that the surface of the m-Em appeared smooth and non-porous, indicating the removal of a substantial amount of the oil among the microspheres. This observation confirmed the capability of ADP to eliminate the oil from the microspheres’ surface. Microspheres of m-EmG with gentamicin were still in an agglomerated state, and the extra phase among m-EmG microspheres was visible after being impregnated with gentamicin. However, no significant statistical difference was found in particle size among the Em, m-Em, and m-EmG groups. The average particle size of the gel microspheres in the Em, m-Em, and m-EmG groups ranged from 22.5, 19.6, and 16.1 μm, but there was no significant difference between the groups (*p* > 0.05).

#### 3.1.2. Absorption IR Spectra

**Figure 2:**
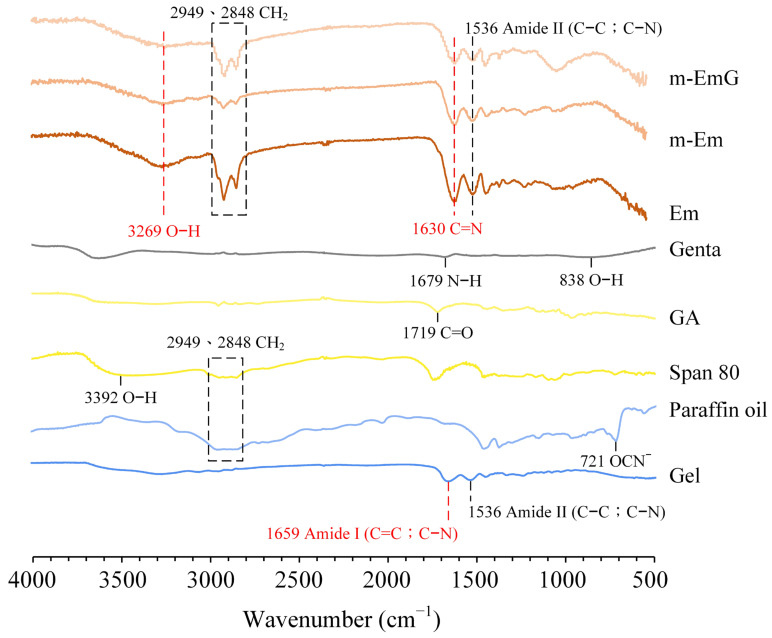
ATR−FTIR spectrum analysis of each raw material (Gel: gelatin, paraffin oil, Span 80, GA: glutaraldehyde, gentamicin) and each emulsified gel microsphere (Em, mEm, and m-EmG).

Based on the infrared spectra (Figure 2), Em, m-Em, and m-EmG microspheres displayed absorption bands, indicating amide II (C–C; C–N) absorption at 1536 cm^−1^ with characteristic C=N at 1630 cm^−1^. These spectral features provide evidence that the gel was effectively cross-linked by GA. The presence of CH_2_ absorption bands at 2848 cm^−1^ and 2949 cm^−1^, along with the smaller CH_2_ absorptions of m-Em compared to Em, indicated the significant removal of the paraffin oil from ADP-modified gel spheres. Gentamicin absorption was found to be insignificant. Observing the hydroxyl (O–H) at 3269 cm^−1^ confirms the successful preparation of emulsified microspheres.

#### 3.1.3. Degradation Measurement of Em and m-Em

**Figure 3:**
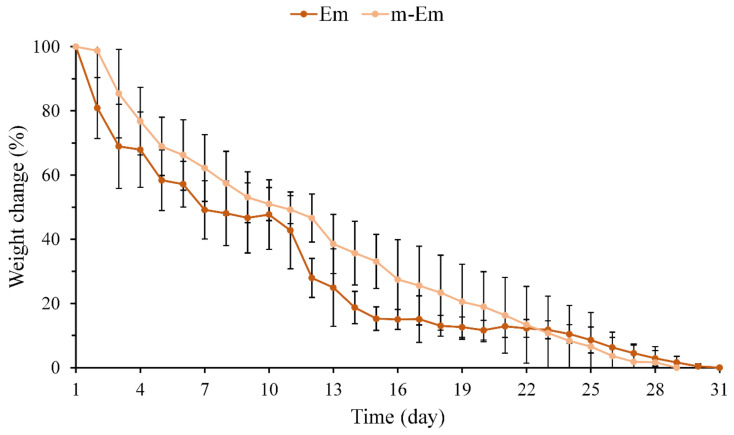
Weight loss curve of hydrogel microspheres Em and m-Em after complete degradation after immersion in diH_2_O (*n* = 10).

The weight loss of hydrogel microspheres Em and m-Em following immersion in diH_2_O was quantified, and the results are presented in Figure 3. No noticeable difference in the weight loss pattern of the emulsified microspheres was observed between pre- and post-modification. The Em group underwent complete degradation after 28 days, whereas the m-Em group exhibited degradation after approximately 31 days. The cross-linking degrees of the two groups by GA were determined to be 86.7 ± 2.5 and 85.1 ± 3.4% (*n* = 6, *p* > 0.05), respectively. These findings collectively indicate that the degradation of Em microspheres remains unaltered following modification.

#### 3.1.4. TGA and DSC Analysis of m-Em and m-EmG

**Figure 4:**
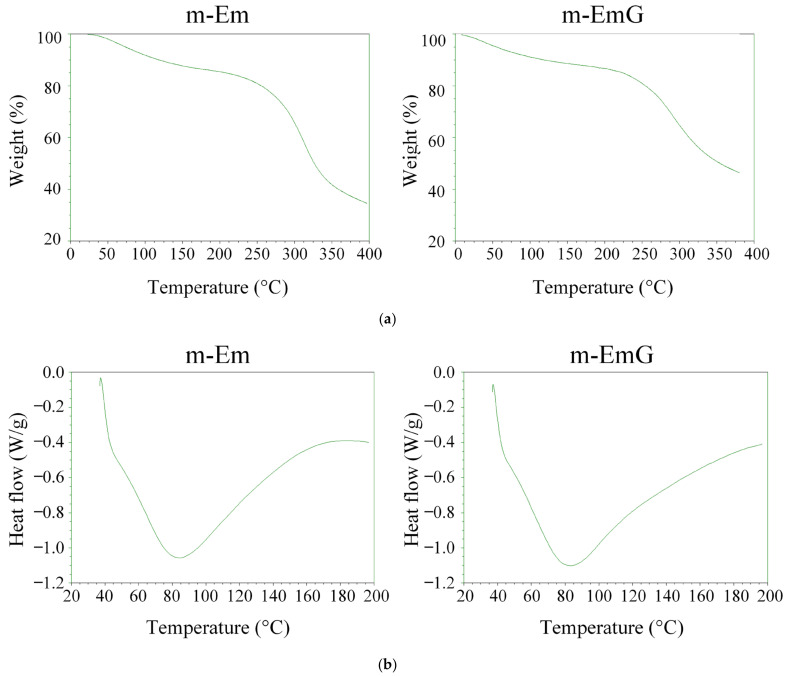
(**a**) TGA and (**b**) DSC analysis of modified gel microspheres (m-Em) and after antibiotic impregnation (m-EmG).

The TGA and DSC results for m-Em and m-EmG are depicted in Figure 4. The variation in cross-linking degree led to a rise in the temperature range for thermal cracking rather than a specific temperature. Notably, no discernible disparity was found before (m-Em) and after gentamicin impregnation (m-EmG). As indicated by the DSC analysis, both groups presented distinct endothermic bands at 80 °C, corresponding to the gel’s glass transition temperature (Tg) post-cross-linking. Typically, the Tg of the gel falls within the range of 30 to 40 °C [35]. These outcomes suggest no substantial differentiation in the thermal characteristics of m-Em and m-EmG. Furthermore, the disparity in DSC was inconsequential, aligning with the findings on cross-linking degrees.

#### 3.1.5. Antibiotic Release of m-EmG

**Figure 5:**
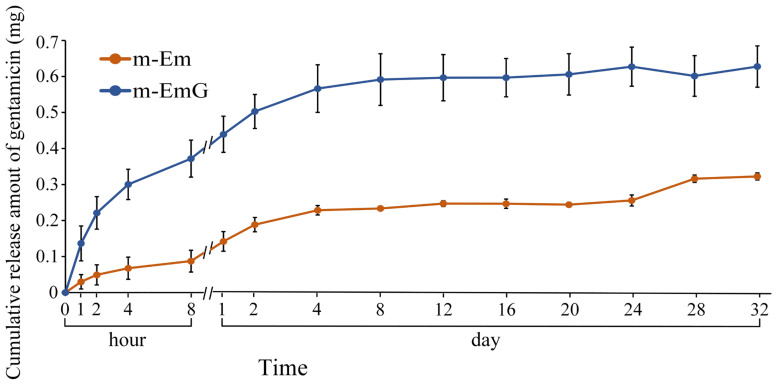
Comparison of gentamicin release from m-EmG after immersion in diH_2_O and m-Em background (*n* = 10).

The drug release profile of gentamicin within the 0.01 g of m-EmG gel microspheres, along with the background value of m-Em, is illustrated in Figure 5. The background value of m-Em should be subtracted from the measured value of m-EmG to obtain the actual drug release measurement. In the m-EmG group, a substantial release of gentamicin occurred approximately 2 days after immersion, transitioning from an initial rapid release to a sustained release pattern. The process involved gel backbone slippage within the microspheres and subsequent degradation of the gel molecules, resulting in sustained release. The release of m-EmG exhibited stability and sustained release relative to the Em value for a period of approximately 2–24 days. The final release was attributed to releasing the remaining bonds within the microspheres after degradation.

#### 3.1.6. Antibacterial Efficiency of m-EmG

**Figure 6:**
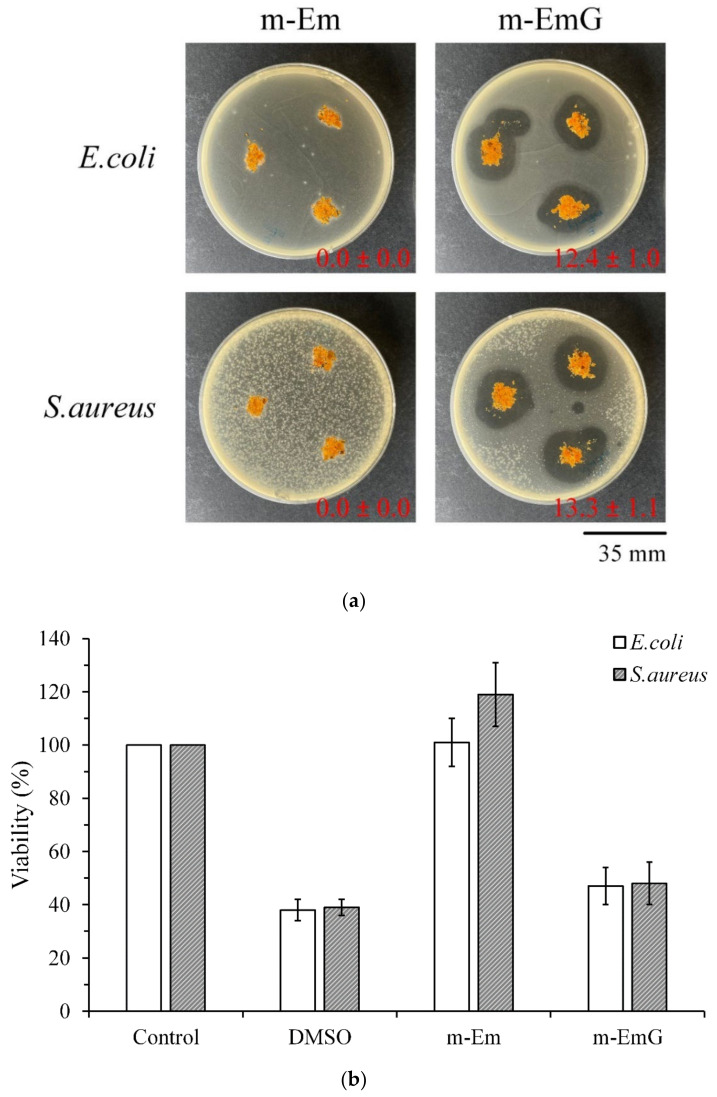
Comparison of antibacterial effects of m-Em and m-EmG against *E. coli* and *S. aureus* after one day of incubation: (**a**) qualitative and (**b**) quantitative (*n* = 3) testing.

To further confirm the antibacterial effect of m-EmG, Figure 6a shows the qualitative antibacterial effect of m-Em and m-EmG on *E. coli* and *S. aureus* after 1 day of culture. m-EmG had an obvious inhibitory zone, while the m-Em group had no inhibition zone, showing the effective impregnation and release of antibiotics in the modified gel microspheres. Figure 6b shows the quantitative antibacterial test, and the results showed that the antibacterial efficiency of the m-EmG group was similar to that of the positive control, DMSO. The m-EmG group demonstrates stronger antibacterial ability and drug release compared with the m-Em group. This finding confirms that the measured gentamicin values in m-Em (Figure 5) should be considered interference background values for the release of paraffin oil.

#### 3.1.7. Biocompatibilities of Em, m-Em, and m-EmG

**Figure 7:**
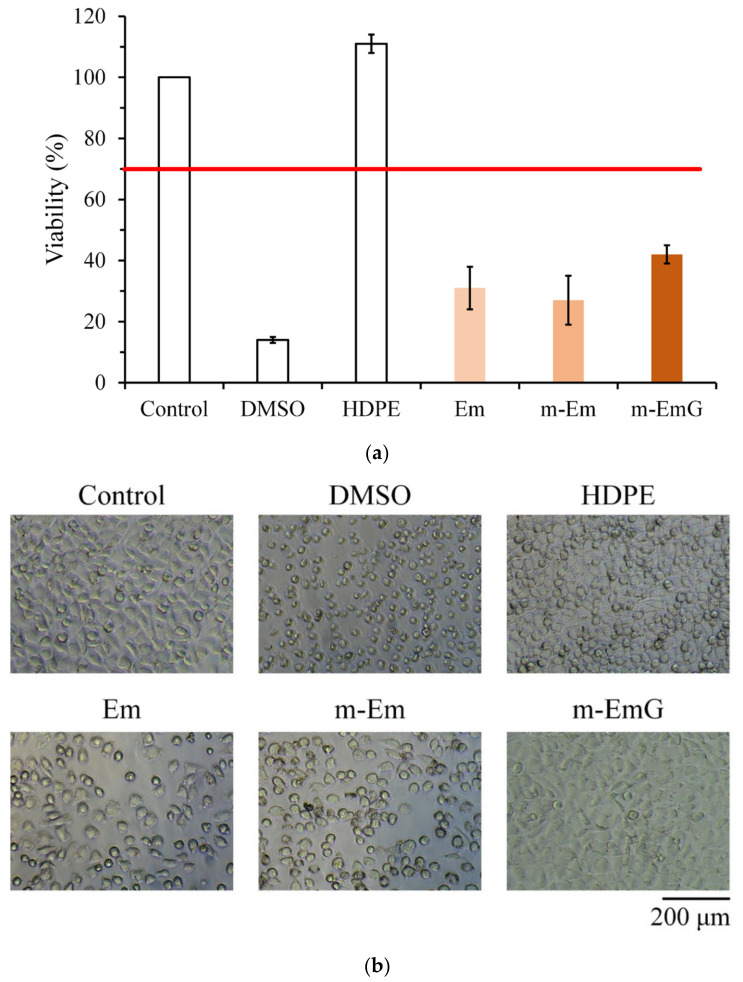
Comparison of cytotoxicity of m-Em and m-EmG extracts after one day of L929 cell culture: (**a**) quantitative (*n* = 6) and (**b**) qualitative testing.

The quantitative cytotoxicity test of each microsphere extract and L929 cells cultured for 1 day is shown in Figure 7a. According to ISO 10993-5, when the cell survival rate is lower than 70% or the cell morphology changes significantly, the sample extract has a toxic effect on the cells [34]. The cell survival rate of the HDPE group was higher than 70%, indicating the effectiveness of sterilization conducted in the study; the cell survival rates after Em, m-Em, and m-EmG modifications were all lower than 70%, which showed that the extract was cytotoxic to L929 cells. This phenomenon could be due to the residual oil phase of paraffin oil, Span 80, or GA.

The cell morphology of extracts cultured with L929 cells for one day is shown in Figure 7b. The appearance of the cells in the control group was plump and spindle-shaped, indicating good cell morphology. However, the different microsphere extracts of Em, m-Em, and m-EmG showed that the cells were shrinking and spherical. The results were similar to those of DMSO, indicating the presence of cytotoxicity. This result is consistent with the quantitative test results.

### 3.2. Characterizations of m-EmG@CPC Composites

#### 3.2.1. IR Spectra of Different Microsphere Ratios of m-EmG@CPC Composites

**Figure 8:**
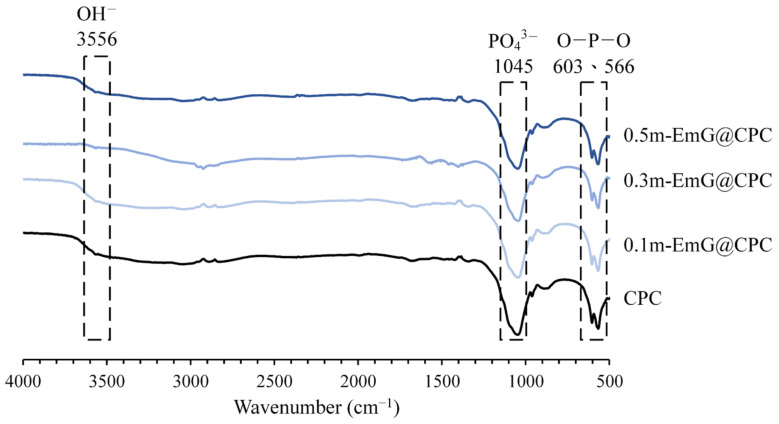
Infrared spectra of different microsphere proportions of m-EmG@CPC composites after soaking in Tris buffer for 1 day.

The FTIR spectra of the m-EmG composite CPC are displayed in Figure 8. The two groups of CPC alone and m-EmG@CPC composites all showed the formation of HA products after immersion. In addition, no difference was found between the spectra, indicating that no new compounds were produced among the m-EmG@CPC composites.

#### 3.2.2. Diffraction Patterns of m-EmG@CPC Composites

**Figure 9:**
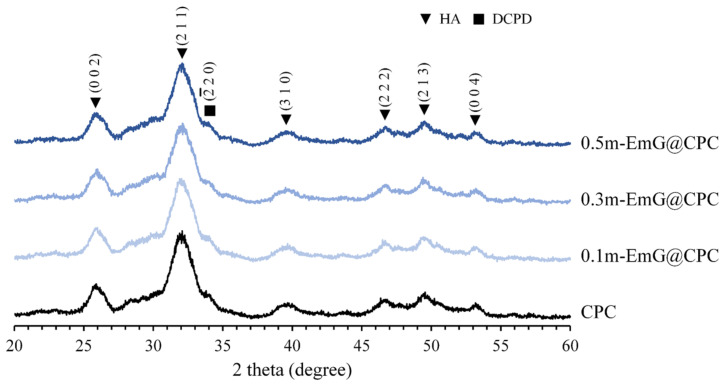
Diffraction patterns of different m-EmG@CPC composites after soaking in Tris buffer for 1 day.

The XRD analysis of m-EmG@CPC composites after soaking in Tris buffer for one day is depicted in Figure 9. Aside from the presence of HA (JCPDS 090432), only a small amount of DCPD peak (JCPDS 010395) diffraction was observed. No discernible differences were found in the diffraction patterns between the groups. This finding was further supported by the FTIR analysis, which demonstrated that the inclusion of m-EmG microsphere composite CPC did not alter the major HA product, nor did it result in the formation of any additional product phases compared with CPC alone.

#### 3.2.3. Injectability and Disintegration of m-EmG@CPC Composites

CPC is in the form of a paste before hardening and can be delivered by injection to repair irregular bone defects in clinical applications. However, if the CPC working or setting time is too short, then it will harden in the delivered tooling and become impossible to inject. In addition, if CPC is exposed to a large amount of blood after injection, then it causes disintegration. This means that its anti-disintegration ability is poor, and its clinical application will be more limited [36].

**Figure 10:**
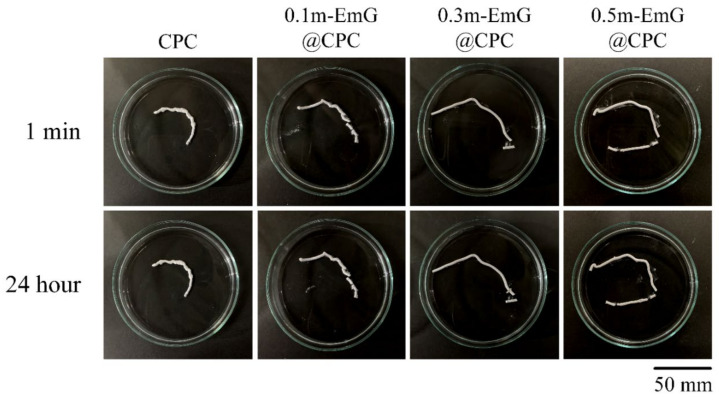
Injectability and disintegration of different m-EmG@CPC composites compared to CPC alone: immediately and after 24 h of immersed observation.

The results of the injectability and disintegration resistance of m-EmG@CPC composites are shown in Figure 10. According to the above-mentioned experimental method, injection of bone cement paste into water, and observation, the residual rate in each group was less than 15%, indicating that each m-EmG@CPC composite had excellent injectability. There was no obvious disintegration after 1 min and 24 h of m-EmG@CPC composites immersed in diH_2_O.

#### 3.2.4. Compressive Strength and Fractural Surface Observation of m-EmG@CPC Composites

**Figure 11:**
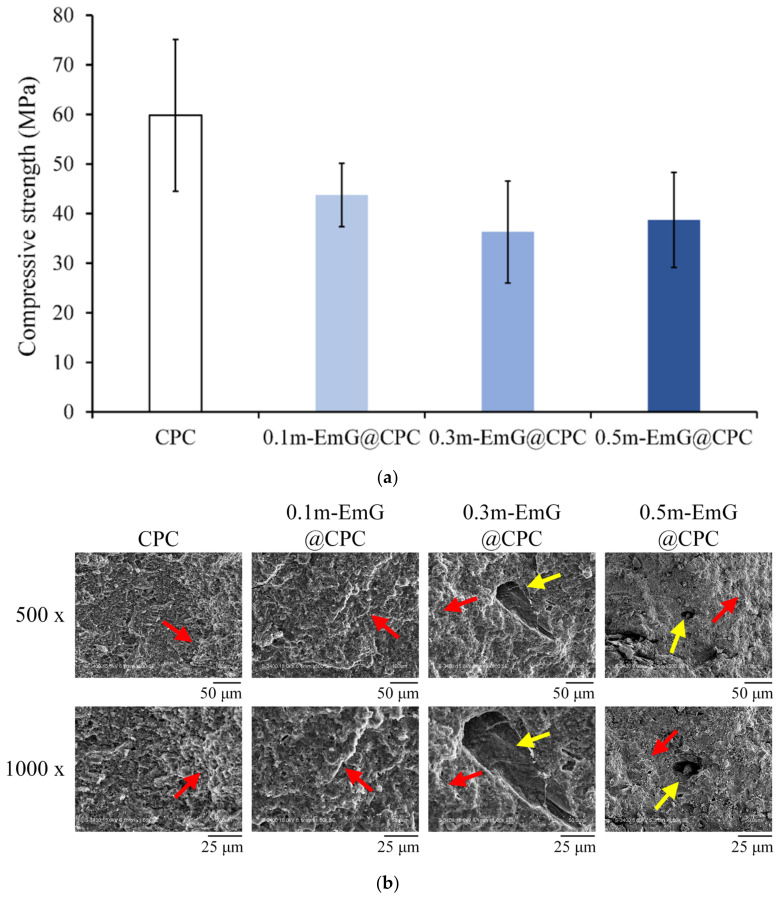
(**a**) compression test (*n* = 10) and (**b**) fracture surface observation of different m-EmG@CPC composites compared to CPC alone after soaking in Tris buffer for 1 day (red arrowhead: HA product phase morphology; yellow arrowhead: pore).

The strength of bone grafts should be at least close to the compressive strength of human trabecular bone, which is approximately 30 MPa [37]. The compression results of the m-EmG@CPC composite after soaking in Tris buffer for one day is shown in Figure 11a. The highest value of CPC alone was 59.82 ± 15.33 MPa. With the addition of m-EmG, the compressive strength showed a downward trend, as CPC is a ceramic material. After adding emulsified microspheres, there will be an interface due to different material properties after compositing. Therefore, the interface generally has large stress in compression and produces cracks, resulting in reduced compressive strength [15,16,38], but there was no statistical significance between m-EmG@CPC composites. The strength of 0.5m-EmG@CPC was 38.74 ± 9.59 MPa, which still meets the clinical application standards.

The microstructure of the fracture surface of m-EmG@CPC post-compression is illustrated in Figure 11b. The presence of the apatite phase after the CPC reaction was consistent across all groups. The red arrow denotes the coral reef-like crystal structure, signifying that the incorporation of m-EmG microspheres with CPC did not impede the reaction of the HA products. Furthermore, conspicuous pores, indicated by yellow arrows, became increasingly evident with the addition of m-EmG microspheres.

#### 3.2.5. Qualitative and Quantitative Antibacterial Testing

**Figure 12:**
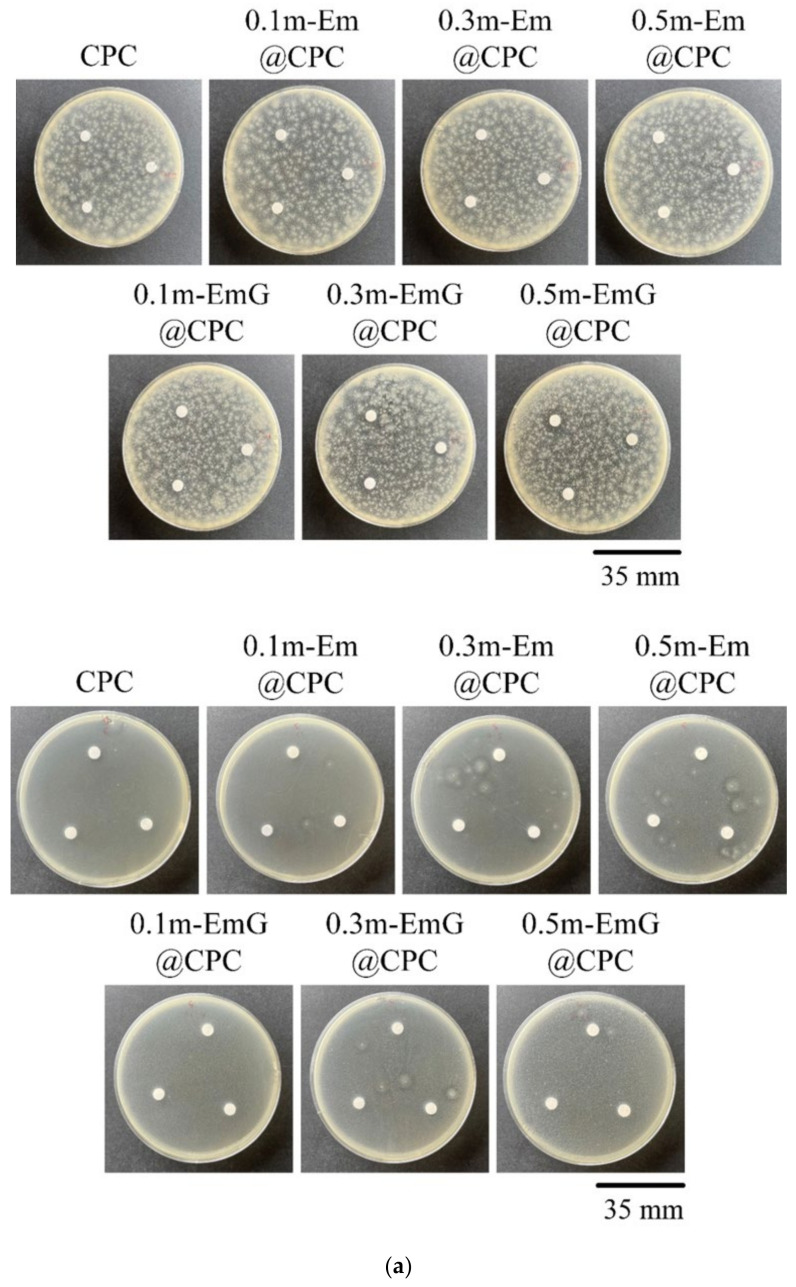

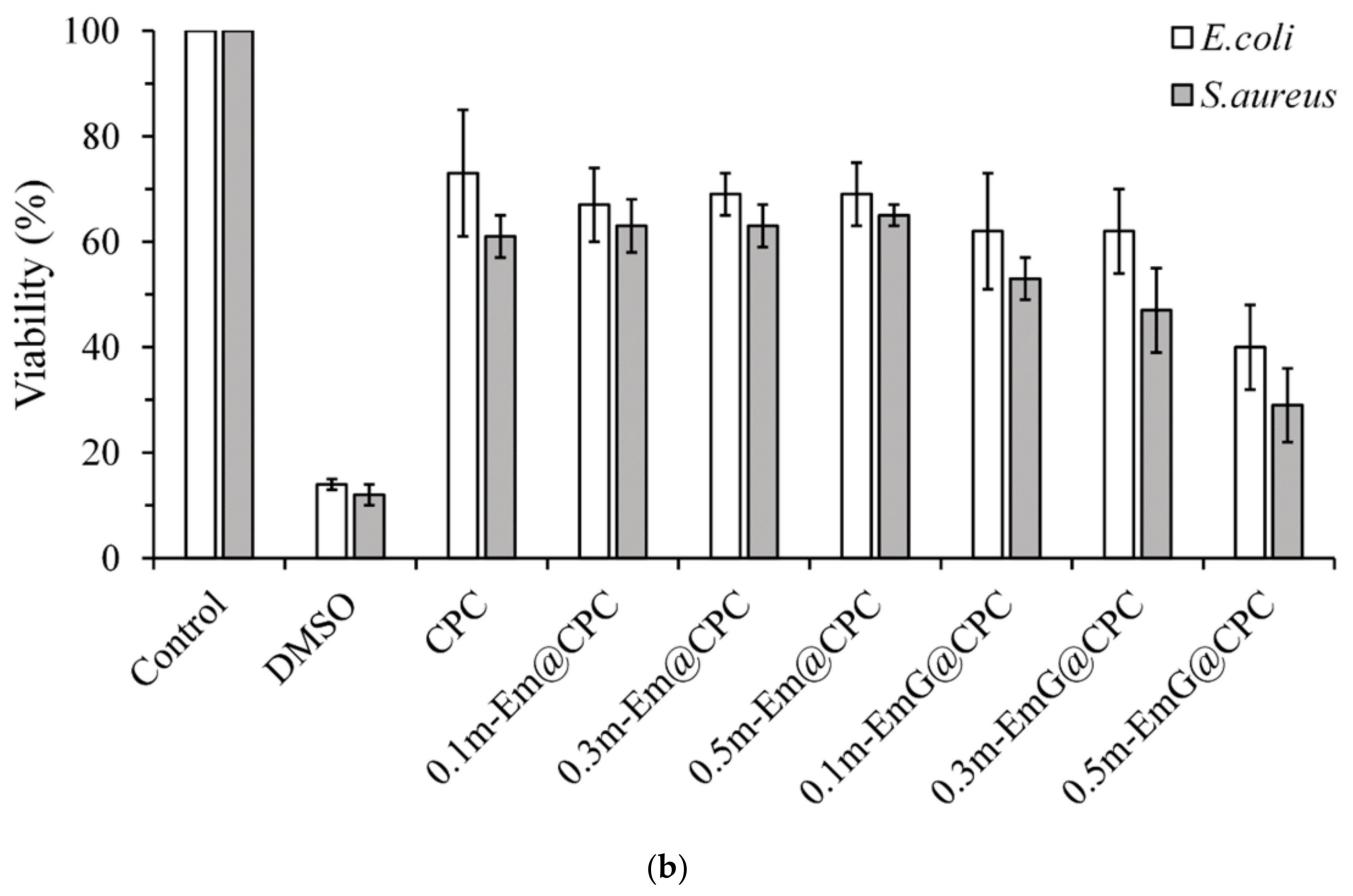
(**a**) Qualitative antibacterial testing of different m-EmG@CPC composites versus CPC alone exposed to *E. coli* and *S. aureus* for one day. (**b**) Quantitative antibacterial testing of different m-EmG@CPC composites compared to CPC alone cultured in *E. coli* and *S. aureus* broths for one day (*n* = 3).

The m-EmG@CPC composites were incubated with *E. coli* and *S. aureus* for 1 day for qualitative and quantitative antibacterial testing (Figure 12). No inhibition zone was produced in the qualitative contact culture, regardless of the gentamicin-containing groups (Figure 12a). The antibiotics in m-EmG microspheres were difficult to diffuse and release.

From the quantitative antibacterial results of *E. coli* and *S. aureus* broth cultured for one day, the antibacterial effect of the 0.5m-EmG@CPC containing gentamicin group was the most obvious, and the effect was enhanced with the increase of m-EmG microspheres. The significantly improved antibacterial ability means that after the gentamicin-loaded gel microspheres composite CPC, spreads, the antibacterial properties can be successfully released, effectively inhibiting *E. coli* and *S. aureus*.

#### 3.2.6. Gentamicin Release of 0.5m-EmG@CPC Composite

**Figure 13:**
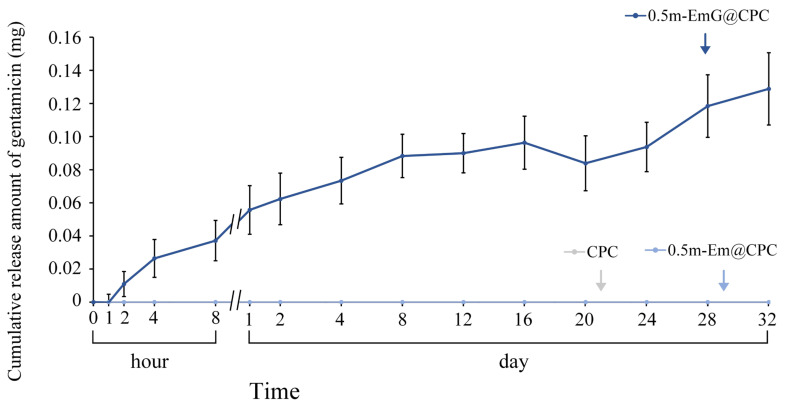
Release curve of gentamicin from the m-EmG@CPC composite immersed in diH_2_O (*n* = 10).

The above tests showed that the 0.5m-EmG@CPC group had the lowest compressive strength required for clinical surgery, and the antibacterial test showed that only this group had apparent antibacterial ability; so, subsequent experiments were all on this 0.5m-EmG@CPC. Compared with 0.5m-Em@CPC (without antibiotics) as a control, none showed antibacterial effects. From Figure 13, the release of gentamicin from 0.5m-EmG@CPC was measured; approximately 0.08 mg of gentamicin was released after 20 days of immersion. The total release amount was significantly lower than that of pure m-EmG gel microspheres, indicating that m-EmG composite CPC could slow down the initial burst release of gentamicin and further sustain release over several days. This phenomenon enables research designs to prevent postoperative bacterial infections.

#### 3.2.7. Working/Setting Times

The working and setting times of each gel microspheres composite CPC are shown in Table 1. The working time was about 5 min, and the setting time was about 10 min. No noticeable difference was found among the groups (*p* > 0.05). For clinical applications, the expected working time is 4 to 10 min, and the appropriate setting time is 10 to 20 min; the results showed that each group in this experiment met this requirement.

### 3.3. In Vitro Cytotoxicity and D1 Cell Interactions

#### 3.3.1. Cytotoxicity

**Figure 14:**
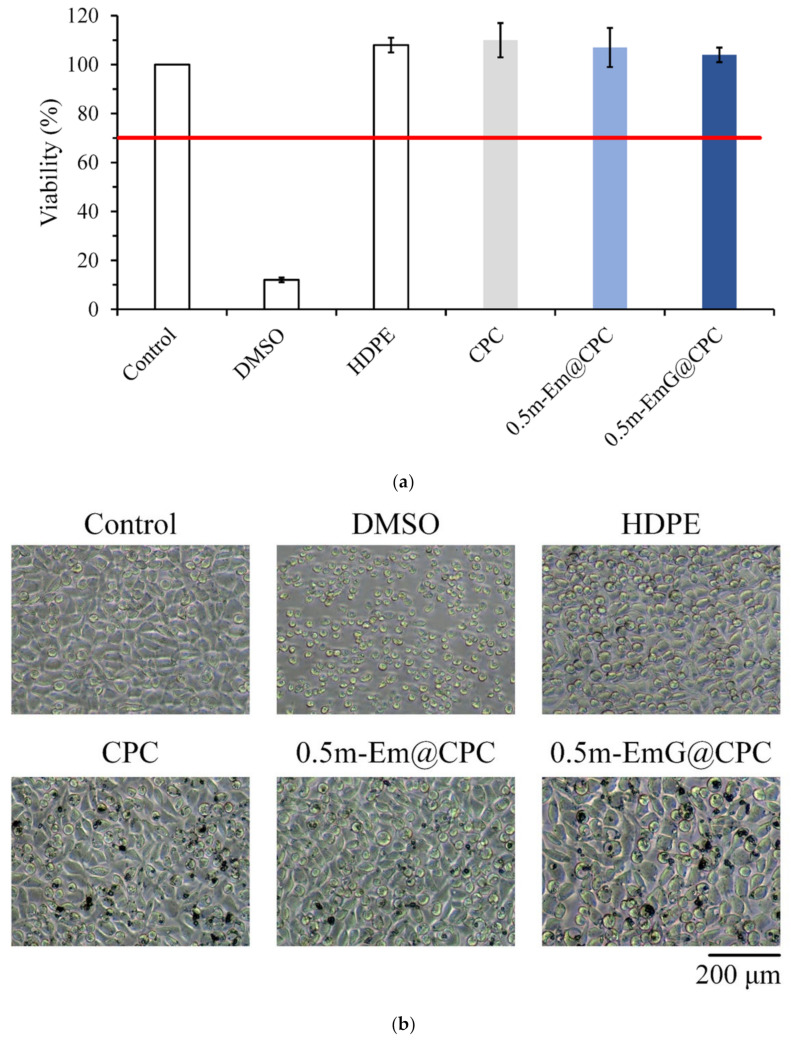
(**a**) quantitative cytotoxicity (*n* = 6) and (**b**) qualitative cell morphology of CPC alone, 0.5m-Em@CPC, and 0.5m-EmG@CPC extracts cultured with L929 cells for one day.

The results in Figure 14a confirmed that the extract of m-EmG@CPC had no cytotoxicity to L929 cells. Cell morphology was observed after one day of culturing L929 with m-EmG@CPC extract. The cells were revealed in a complete spindle shape, indicating that they were in good shape. Therefore, m-EmG@CPC had no cytotoxicity.

#### 3.3.2. Osteoprogenitor-D1 Cell Proliferation and Mineralization

**Figure 15:**
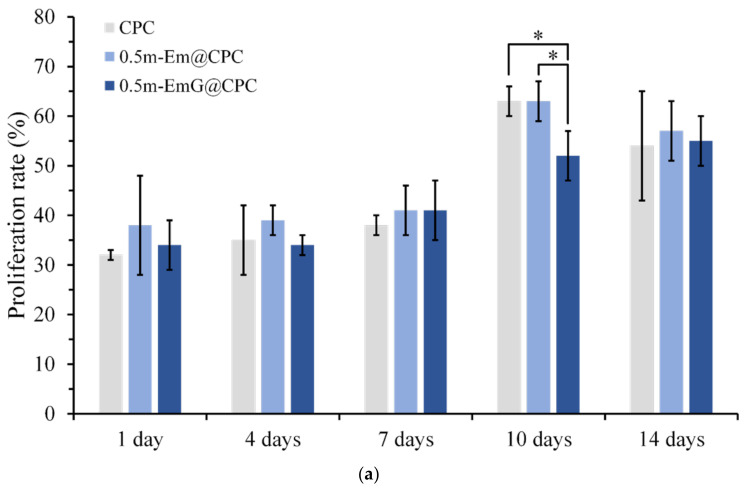

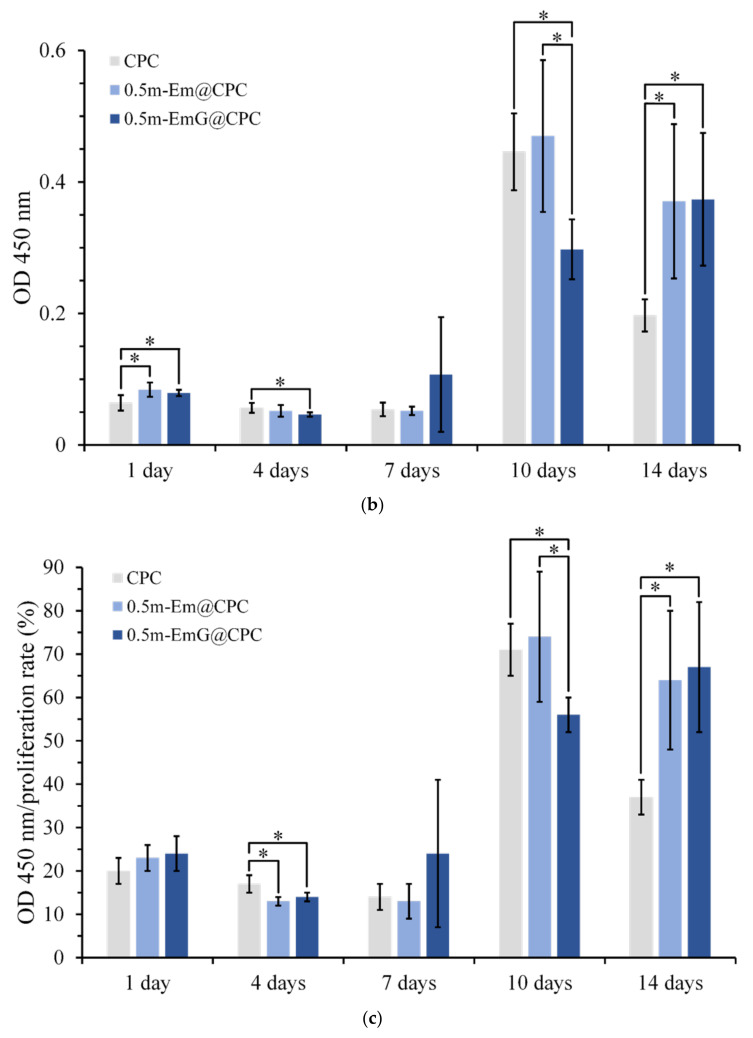

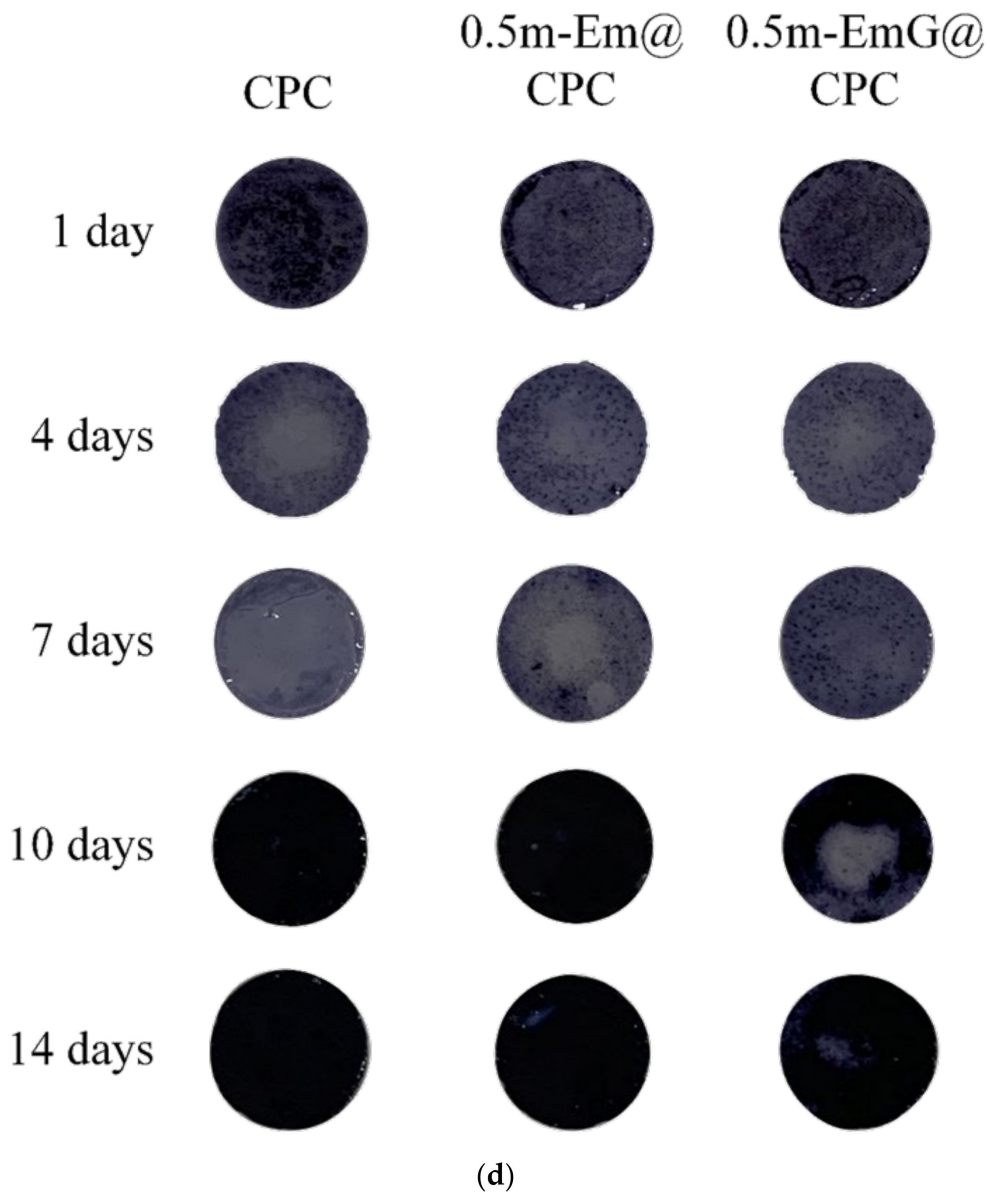
(**a**) Proliferation of CPC alone, 0.5m-Em@CPC, and 0.5m-EmG@CPC composites cultured with D1 cells for 14 days. The symbol * indicates a significant difference between groups based on a one-way ANOVA (*p* < 0.05, *n* = 3). (**b**) ALP secretion (OD_450_) of CPC alone, 0.5m-Em@CPC, and 0.5m-EmG@CPC composites cultured with D1 cells for 14 days; symbol * indicates a significant difference between groups based on one-way ANOVA (*p* < 0.05, *n* = 3). (**c**) Semi-quantitative analysis of ALP secretion of CPC alone, 0.5m-Em@CPC, and 0.5m-EmG@CPC composites cultured with D1 cells for 14 days. The symbol * indicates a significant difference between groups based on a one-way ANOVA (*p* < 0.05, *n* = 3). (**d**) Qualitative ALP staining of CPC alone, 0.5m-Em@CPC, and 0.5m-EmG@CPC composites cultured with D1 cells for 14 days.

The proliferation ability of D1 cells during the period of 14 days was measured by detecting CPC alone and gel microspheres@CPC with or without impregnated gentamicin in contact with D1 cells for 14 days. The comparison results with the blank group are shown in Figure 15a, and cell proliferation increased with time. On day 14, all groups began to reach the plateau phase and no longer proliferated, indicating that osteoprogenitor-D1 cells began mineralizing. Observation of cells on the 10th day showed that when CPC alone and 0.5m-EmG@CPC were compared, cell proliferation was significantly greater than that of 0.5m-EmG@CPC, indicating that the release of gentamicin had a certain negative impact on D1 cells.

ALP is a phosphatase produced by bone cells. It can help hydrolyze phosphate monoesters in the body. When bone cells enter mineralization, they secrete ALP in large quantities and release phosphates to participate in bone matrix construction [16]. In order to confirm the effect of m-EmG@CPC on the mineralization ability of bone cells, the ALP secretion ability can be measured as a mineralization index. As shown in Figure 15b, a small amount of ALP was secreted on days 1, 4, and 7. The ALP secretion of the 0.5m-EmG@CPC group reached its maximum on the 14th day of culture. Figure 15c shows a semi-quantitative analysis of ALP, where the relative ability of a single cell to secrete ALP is obtained by dividing ALP production by the number of cells. The changing trend was the same as that of ALP secretion in Figure 15b. The ALP secretion of the D1 cells cultured on 0.5m-EmG@CPC reached its highest on the 14th day, but the peak time was delayed compared with the 10 days culture on CPC alone and 0.5m-Em@CPC. Overall, gentamicin released from 0.5m-EmG@CPC did not significantly affect the proliferation or ability of D1 cells to secrete ALP, except for the delayed effect.

## 4. Conclusions

Emulsified gel microspheres were prepared, and ADP greatly removed paraffin oil. The drug release and antibacterial tests showed that gentamicin was successfully impregnated into m-EmG microspheres. The total degradation time of m-EmG reached 30 days, and gentamicin was released slowly and sustainably, but m-EmG microspheres were still cytotoxic to L929 cells. Obvious pores were found inside the m-EmG@CPC after composite immersion and compression, although adding gel microspheres did not hinder the product phase HA generation. From the mechanical tests, the compressive strength of m-EmG@CPChe decreased but still met clinical requirements. No obvious differences were detected compared with CPC alone, especially in terms of working/setting, injectability, and disintegration resistance in operations. For antibacterial activity and drug release, m-EmG@CPC was observed to effectively release gentamicin, resulting in antibacterial effects against *S. aureus* and *E. coli*. From in vitro cell testing, the maximum addition group of 0.5 wt.% m-EmG composite CPC was not cytotoxic and had no negative impact on the proliferation of D1 cells; hence, the mineralization of ALP secretion with CPC alone confirmed comparability. Based on the results, 0.5m-EmG@CPC was selected as the best group and is expected to become a new composite bone cement choice in future bone restorations and generations.

## Figures and Tables

**Table 1:** Working and setting times of CPC alone, m-Em@CPC, and m-EmG@CPC (*n* = 10).

Samples	Working Time (min)	Setting Time (min)
CPC	5.18 ± 0.09	10.26 ± 0.12
0.5m-Em@CPC	5.24 ± 0.08	10.29 ± 0.03
0.5m-EmG@CPC	5.24 ± 0.09	10.30 ± 0.07

## Data Availability

The original contributions presented in the study are included in the article, further inquiries can be directed to the corresponding authors.
